# Researchers’ views on and practices of knowledge translation: an international survey of transfusion medicine researchers

**DOI:** 10.1186/s43058-024-00546-3

**Published:** 2024-01-12

**Authors:** Amanda Thijsen, Barbara Masser, Tanya Ellen Davison, Anna Williamson

**Affiliations:** 1 School of Public Health, The University of Sydney, Sydney, Australia; 2 Research & Development, Australian Red Cross Lifeblood, Sydney, Australia; 3 School of Psychology, The University of Queensland, Brisbane, Australia; 4 Research & Development, Australian Red Cross Lifeblood, Brisbane, Australia; 5 Research & Innovation, Silver Chain, Melbourne, Australia; 6 Monash Art, Design and Architecture, Monash University, Melbourne, Australia

**Keywords:** Knowledge translation, Researchers, Transfusion medicine, Blood, End-user engagement, Dissemination, Diffusion, Implementation science, Knowledge mobilisation

## Abstract

**Background:**

Health research is often driven by the desire to improve the care and health of the community; however, the translation of research evidence into policy and practice is not guaranteed. Knowledge translation (KT) activities, such as dissemination and end-user engagement by researchers, are important to achieving this goal. This study examined researchers’ views on and practices of KT in the field of transfusion medicine.

**Methods:**

An anonymous, cross-sectional survey was distributed to transfusion medicine researchers in May 2022 by emailing corresponding authors of papers in four major blood journals, emailing grant recipients, posting on social media, and through international blood operator networks. Comparative analyses were conducted for career stage, work setting, research type, and KT training.

**Results:**

The final sample included 117 researchers from 33 countries. Most participants reported that research translation was important (86%) and felt it was their responsibility (69%). Fewer than half felt they had the skills to translate their research (45%) or knew which strategies to employ (45%). When examining how research findings are shared, most reported using diffusion activities (86%), including publishing in peer-reviewed journals (74%), or presenting at academic conferences (72%). Fewer used dissemination methods (60%), such as developing educational materials (29%) or writing plain language summaries (30%). Greater use of tailored dissemination strategies was seen among researchers with KT training, whilst traditional diffusion strategies were used more by those working in an academic setting. Most participants had engaged end-users in their research (72%), primarily to consult on a research component (47%) or to involve them in the research process (45%). End-user engagement was greater among researchers with established careers, working in both academic and applied settings, and with KT training.

**Conclusions:**

Whilst participating researchers acknowledged the importance of KT, they typically focused on traditional diffusion strategies. This is despite well-established knowledge of the limited impact of these strategies in achieving KT. Those with KT training were more likely to use tailored dissemination strategies and engage end-users in their research. This demonstrates the value of sharing knowledge from the KT field with health researchers to facilitate KT.

**Supplementary Information:**

The online version contains supplementary material available at 10.1186/s43058-024-00546-3.

Contributions to the literature

- This study showed that whilst researchers feel responsible for knowledge translation (KT), many do not feel they have the skills or knowledge to effectively translate their research.
- Traditional diffusion strategies remain the most common ways to share research knowledge in transfusion medicine.
- The findings of this paper showed differences in KT practices by career stage, work setting, and self-reported KT training
- This indicates the potential for KT training to increase the use of tailored dissemination strategies and end-user engagement among researchers.

## Background

Offering the best possible care, improving the lives of the community, and contributing to the broader scientific knowledge are some of the key motivators for conducting health-related research [1, 2]. Ensuring knowledge gained from research is appropriately disseminated and/or translated is vital to achieving this goal. This process is often labelled as knowledge translation (KT) and is defined as “the dynamic and iterative process that includes the synthesis, dissemination, exchange, and ethically sound application of knowledge to improve health, provide more effective health services and products, and strengthen the healthcare system” (p.4) [3]. Knowledge producers, such as researchers, play a central role in this process. They can influence the dissemination of knowledge through how the findings are presented and communicated and through the selection of target audiences [4, 5]. Researchers can apply passive and untargeted strategies, such as publishing in peer-reviewed journals, mass mailings, or conference presentations. They can also apply more active and tailored strategies, such as plain language summaries, patient decision support aids, and interactive small group meetings with end-users [6–8].

Whilst these dissemination strategies are necessary to spread information, they are not sufficient to ensure actual use of knowledge [5, 9]. Therefore, in addition to disseminating research findings, researchers are encouraged to involve research end-users, such as policymakers and practitioners, throughout the entire research process. The goal of end-user engagement is to increase the relevance of the research as well as to improve the accessibility, appropriateness, and understandability of the research evidence [5, 10]. In order to achieve these goals, it is important to establish meaningful and active collaborations between researchers and end-users in determining research priorities, conducting the research, interpreting outcomes, and translating findings into policy and practice [10, 11]. An essential step in minimising the knowledge-to-practice gap is gaining an understanding of how researchers disseminate and engage end-users in their research.

A number of studies have investigated how researchers facilitate the KT process through dissemination and end-user engagement. A survey [12] conducted in 2001 among health researchers in Alberta, Canada, tried to provide objective measures of passive strategies—by summing the number of publications in the last five years—and active strategies—by summing the number of plain language reports and the number of times they involved end-users in their research. The authors found that researchers reported more passive than active dissemination of their research, with this particularly evident among basic science researchers. Similar findings have emerged in other surveys of researchers working in health-related fields, with more reporting using mostly passive diffusion strategies, including academic journals (88–99%) and academic conferences (90–93%) than active tailored approaches, such as plain language summaries (33–64%) and face-to-face meetings (48–68%) [13–16]. A 2012 survey study found that only one-third of US-based health researchers involved end-users in their research [17]. Consistent with this, a more recent international survey found that involving end-users was the least employed KT strategy of authors of public health trial publications [15]. A more in-depth exploration [11] showed that health researchers in Canada mostly engaged end-users in their research by informing them about their findings or by getting their feedback on certain aspects of the research. Only a few actively collaborated with end-users throughout the research process. The authors also reported that researchers believed that some basic and biomedical research areas were not appropriate for engagement throughout the research process with end-users such as patients and the public.

These results highlight the knowledge-to-practice gap that the field of KT faces. Studies have shown that the effective use of KT activities is associated with a greater impact of the research on public health policy and practice [15, 18]. In particular, disseminating study findings and providing training to end-users on how to use the intervention have been identified as the most effective KT strategies in ensuring the translation of trial findings [15]. Despite the importance of dissemination and end-user engagement activities, there is a lack of understanding of whether these activities are influenced by certain characteristics of the knowledge being produced and the person conducting the research, such as the career stage of the researcher, the setting in which the researcher is based, the type of research conducted, and whether the researcher has been trained on KT. Whilst it has been suggested that these characteristics can influence KT activities [19, 20], this has not been thoroughly investigated to our knowledge. This is an important knowledge gap as understanding these factors can help future efforts to improve KT.

The aim of this international survey study is to examine researchers’ views on and practices of two aspects of KT (dissemination and exchange) in the field of transfusion medicine. This is a multidisciplinary field focusing on the collection, storage, and use of blood and blood-related products [21, 22]. Transfusion medicine includes basic science research, such as investigations into reducing viral transmission of blood products, treatment methods using blood-related products, and optimal storage solutions of blood and blood components. It also includes applied science research, which focuses on blood donor management such as increasing blood donor recruitment and retention and reducing adverse events in relation to the collection of blood [22, 23]. Research conducted in this area is driven by gaps in knowledge and operational needs. Researchers can be based in an applied setting, such as a blood collection agency or a hospital, and/or an academic setting, such as a university or research institute [22]. A recent review of the published literature showed that, whilst there is some evidence of KT practices in transfusion medicine, it is in the early stages [24]. Further, researchers in this field are faced with similar KT barriers as others, such as lack of time, funding, and/or resources. They also perceive maintaining good relationships with end-users as critical to the KT process [25]. We extend these findings by examining researchers’ KT activities in the area of transfusion medicine. Specifically, our study objectives were to examine (1) transfusion medicine researchers’ views of and attitudes towards KT, (2) their knowledge dissemination activities, and (3) their end-user engagement activities. We examined the differences by career stage, work setting, research type, and KT training. Documenting these views and activities by researchers is important to gain an understanding of how to minimise the knowledge-to-practice gap in transfusion medicine.

## Methods

This paper presents a component of a larger cross-sectional survey study on KT in transfusion medicine that was conducted with an international cohort of researchers. Data were collected and managed using REDCap electronic data capture tools hosted at the University of Sydney. Participants were recruited through five main strategies using a combination of direct emails to corresponding authors of published articles in well-known transfusion medicine journals and grant recipients of research relating to transfusion medicine (*n* = 1645), distribution via an international blood operator network, and public social media posts in May 2022, with details published elsewhere [25]. Participants were excluded from participating in the study if they indicated in the screening question that they did not spend any of their working time on research activities. Ethical approval to conduct the study was obtained from the University of Sydney (#2021/854). The STROBE Checklist [26] was used to guide our reporting (see Additional file 1).

### Survey instrument

The questionnaire was developed using existing literature on KT activities and end-user research engagement [6, 11, 27, 28]. Feedback was sought on the wording of the questions and survey flow from three individuals working in transfusion medicine as a researcher or medical officer.

The questionnaire consisted of several sections. First, participants were asked a range of demographic and work-related questions including gender, country currently based, primary and secondary work setting, current type of research methodology being used, years active in transfusion medicine, and whether they have ever received training on KT. The second part focused on dissemination activities whereby participants were asked “To what extent do you do the following activities to disseminate your research findings?”, rating 11 activities on a 5-point Likert scale (1 = never, 5 = always). The list of dissemination activities was informed by Lomas’ taxonomy [6] and the Guide to Knowledge Translation Planning at CIHR [27]. The third part of the questionnaire focused on end-user engagement activities informed by Crockett et al. [11] and included multiple-choice questions on the level of end-user engagement in general (“At what level have you engaged end-users in your research?”), identifying which end-user groups they have ever involved in their research (“Who have you engaged in the research process?”), and at what research stage (“Please indicate those research phases where you have experience engaging with end-users.”). The final part of the survey elicited participants’ views about who should be responsible for and the importance of KT using 12 statements informed by Lynch et al. [28] that participants responded to on 5-point Likert scales (1 = strongly disagree, 5 = strongly agree). Survey questions are available in Additional file 2.

### Statistical analysis

For descriptive analyses, adopting the approach taken by Lynch et al. [28], responses to the statements on the importance of KT on 5-point Likert scales were collapsed into three categories as affirmative (strongly agree, agree), neutral (neutral), and not affirmative (disagree, strongly disagree). Similarly, responses to diffusion and dissemination activities given on 5-point Likert scales were categorised as never, rarely/occasionally, and frequently/always for ease of interpretation. In addition, end-user groups were combined as blood donors/recipients (blood donors, blood recipients), front-line staff (blood collection staff, blood processing staff, hospital staff), senior management/policymakers, general public, and others.

For comparative analyses, responses to primary and secondary work settings were collapsed to create a new variable “work setting”, with the categories “academic” (university and/or research institute), “applied” (government department/agency, blood collection agency, hospital setting, and/or other healthcare service), and “joint” (university/research institute and government department/blood collection agency/hospital setting/other healthcare service). Further, participants’ “research type” was derived from data on research methods with the categories “basic science” (animal studies and/or biospecimen analysis research) and “applied science” (all remaining categories). The career stage was derived from years active in transfusion medicine, with the categories “early/mid-career” (1–15 years) and “established career” (16 years and over). Finally, “KT training” was dichotomised as yes or no, with no comprising responses of “no” and “don’t know/unsure”.

Sample characteristics and responses to survey items are described using medians (interquartile range) and means (standard deviation) for continuous variables and by frequencies (percentages) for categorical variables. Differences between career stage, work setting, research type, and KT training were investigated using independent *t*-tests, chi-squared tests, and one-way analysis of variance, with significant effects further investigated using Tukey’s HSD tests. All analyses were performed using statistical software (IBM SPSS Statistics 28.0; IBM Corporation) with statistical significance defined as *p* < 0.05.

## Results

A total of 131 people responded to the survey. However, 10% (*n* = 13) did not complete the relevant survey sections, and one participant indicated not conducting research, leaving 117 eligible responses available for analysis. Table 1 shows the characteristics of the final sample. Participants were diverse in gender, with approximately equal numbers of men and women, and diverse in their work setting, with 41% indicating working in two different settings. When combining the two types of work settings, 23% worked solely in an academic setting, 48% worked solely in an applied setting, and 28% worked in a joint setting. Participants also used a wide variety of research methods, with 33% using at least one basic science method. Further, participants were quite experienced, with 43% having worked in the area of transfusion medicine for more than 15 years (range 1–50 years). The sample included participants from 33 countries, including Australia, the USA, the Netherlands, Canada, the UK, Cameroon, Argentina, Saudi Arabia, and South Korea.

**Table 1:** Participant characteristics (*n* = 117)^a^

Variables	*n* (%)
*Gender*	
Man/male	58 (49.6)
Woman/female	57 (48.7)
Non-binary	1 (0.9)
Prefer not to say	1 (0.9)
*Main work setting*	
University	28 (23.9)
Research institute	11 (9.4)
Government department or agency	2 (1.7)
Blood collection agency	36 (30.8)
Hospital setting	32 (27.4)
Healthcare service (other)	1 (0.9)
Other	6 (5.1)
Missing	1 (0.9)
*Secondary work setting*	
University	24 (20.5)
Research institute	2 (1.7)
Government department or agency	7 (6.0)
Blood collection agency	7 (6.0)
Hospital setting	8 (6.8)
None	65 (55.6)
Missing	4 (3.4)
*Type of methods (MC)*	
Animal studies	11 (9.4)
Biospecimen analysis research	39 (33.3)
Data linkage research	33 (28.2)
Epidemiological research	48 (41.0)
Interventional/clinical trials research	39 (33.3)
Qualitative research	46 (39.3)
Quantitative research	53 (45.3)
Other	17 (14.5)
*Career stage*	
Years active in transfusion medicine	16.6 (± 10.5)
Early to mid-career (1–15 years)	63 (53.8)
Established (16–50 years)	50 (42.7)
Not specified	4 (3.4)
*Knowledge translation training*	
Yes	37 (31.6)
No	69 (59.0)
Unsure/do not know	11 (9.4)

^a^Years active in transfusion medicine presented as mean (standard deviation)

*MC* multiple choice

### Importance, ability, and responsibility for knowledge translation

Researchers’ views on the importance of and responsibility for KT are presented in Table 2. Most participants felt that translating their research is important, and only a few reported that their research is not the sort that can be translated. KT was seen by most participants as the responsibility of clinicians (70%), with fewer attributing KT’s responsibility to researchers (58%). When cross-tabulating these two items, half of the sample (51%) indicated that both clinicians and researchers are responsible for KT, with one quarter reporting it was the responsibility of clinicians only (23%), and smaller numbers indicating KT is the responsibility of researchers only (10%), or neither agreeing nor disagreeing with both statements (16%). However, when asked about their own role, two-thirds of participants felt it was their responsibility to translate their research, with only a few transferring this responsibility to someone else in their team. Despite this sense of responsibility, a third of the participants felt that spending time on KT would take them away from their research. Less than half of the sample reported knowing which strategies to use or felt that they had the skills to translate their research. When looking at KT supports, only a small proportion of the sample reported that adequate funding was available to support KT. Further, most participants agreed that specialised implementation researchers should translate their research and that every research team should include such a researcher.

**Table 2:** Researchers’ views on the importance and responsibility of knowledge translation (*n* = 117)^a^

Statement	Median (IQR)	Level of agreement, *n* (%)			
		**Disagree**	**Neutral**	**Agree**	***Missing***
1. It is important to me that my research is translated	5 (4–5)	0	11 (9.4)	100 (85.5)	6 *(5.1)*
2. My research is not the sort of research that can be translated	2 (1–2)	97 (82.9)	10 (8.5)	4 (3.4)	*6 (5.1)*
3. It is my responsibility to ensure that my research is translated	4 (3–4)	10 (8.5)	19 (16.2)	81 (69.2)	*7 (6.0)*
4. Research translation is the responsibility of someone else in my team	3 (2–3)	53 (45.3)	43 (36.8)	13 (11.1)	*8 (6.8)*
5. Researchers should be responsible for translating research findings into practice	4 (3–4)	10 (8.5)	33 (28.2)	68 (58.1)	*6 (5.1)*
6. Clinicians should be responsible for translating findings into clinical practice	4 (3–4)	5 (4.3)	23 (19.7)	82 (70.1)	*7 (6.0)*
7. I know which strategies should be used (by myself/others) to translate my research	3 (3–4)	26 (22.2)	31 (26.5)	53 (45.3)	7 *(6.8)*
8. I have the skills to ensure my research is translated	3 (3–4)	25 (21.4)	31 (26.5)	53 (45.3)	*8 (6.8)*
9. There is adequate funding to support translation of research	2 (2–3)	71 (60.7)	26 (22.2)	12 (10.3)	*8 (6.8)*
10. Spending time on translating my research would take me away from research (or other work-related activities) I enjoy	3 (2–4)	44 (37.6)	27 (23.1)	37 (31.6)	*9 (7.7)*
11. Researchers with experience/interest in implementation should translate my research	4 (3–4)	6 (5.1)	29 (24.8)	75 (64.1)	*7 (6.0)*
12. Every research team should include a researcher with expertise in implementation	4 (3–4)	11 (9.4)	25 (21.4)	75 (64.1)	*6 (5.1)*

^a^Rated as strongly disagree (1) to strongly agree (5). For frequencies, “agree”, “strongly agree”, “disagree”, and “strongly disagree” pooled together

Significant differences were found in perceived importance, ability, and responsibility for KT by career stage, research type, work setting, and KT training. Participants differed in their perceived ability to engage in KT, with more experienced researchers reporting knowing which strategies to use (3.58 ± 0.77 vs. 3.03 ± 1.03, *t*(104) = − 3.07, *p* = 0.003) and having the skills to ensure research is translated (3.47 ± 0.75 vs. 3.07 ± 1.09, *t*(100.44) = − 2.22, *p* = 0.029), to a greater extent than less experienced researchers. In addition, researchers with KT training reported significantly greater scores on knowledge of KT strategies (3.72 ± 0.70 vs. 3.05 ± 0.98, *t*(92.72) = 4.10, *p* < 0.001), and perceived KT skills (3.58 ± 0.73 vs. 3.05 ± 1.01, *t*(107) = 2.79, *p* = 0.006), than researchers not reporting any KT training. Further, basic science researchers reported greater KT skills than applied science researchers (3.51 ± 0.80 vs. 3.12 ± 1.02, *t*(103) = 2.05, *p* = 0.043). A significant difference was found in clinician responsibility of KT by work setting, *F*(2,106) = 3.10, *p* = 0.049, with researchers working in a joint work setting more likely to report KT as the responsibility of clinicians than researchers in an academic work setting (4.06 ± 0.72 vs. 3.56 ± 0.65, *p* = 0.039) and researchers working an applied setting not being significantly different from other groups (3.88 ± 0.83, both *p*’s > 0.05). Finally, more experienced researchers reported greater funding to support KT than less experienced researchers (2.60 ± 0.96 vs. 2.09 ± 0.81, *t*(92.15) = − 2.95, *p* = 0.004).

### Dissemination activities

Examining how research findings are shared (see Table 3), most researchers used diffusion activities “frequently” or “always” (86%), with most publishing in peer-reviewed journals and presenting at academic conferences. Researchers reported using more active dissemination activities to a slightly lesser extent (60%), with the most frequently used methods being plain language summaries, new educational materials, or interactive small group meetings/workshops.

**Table 3:** Diffusion and dissemination activities (*n* = 117)^a^

Activities to disseminate research findings	Median (IQR)	Level of engagement, *n* (%)			
		**Never**	**Rarely/occasionally**	**Frequently/always**	***Missing***
*Diffusion activities*					
Publishing in peer-reviewed journals	4 (3–5)	3 (2.6)	28 (23.9)	86 (73.5)	*–*
Presenting at an academic conference	4 (3–5)	2 (1.7)	31 (26.5)	84 (71.8)	*–*
Detailed research reports	3 (2–4)	12 (10.3)	53 (45.3)	50 (42.7)	*2 (1.7)*
*Dissemination activities*					
Developing new educational materials	3 (2–4)	12 (10.3)	70 (59.8)	34 (29.1)	*1 (0.9)*
Writing plain language summaries	3 (2–4)	10 (8.5)	71 (60.7)	35 (29.9)	*1 (0.9)*
Organising an interactive small group meeting/workshop	3 (2–4)	13 (11.1)	71 (60.7)	32 (27.4)	*1 (0.9)*
Preparing a policy or an evidence brief and disseminating it to relevant audiences (e.g. policymakers, health service providers, or administrators)	3 (2–3)	12 (10.3)	83 (70.9)	21 (17.9)	*1 (0.9)*
Creating networks or networking with end-users such as policymakers and practitioners (e.g. give presentations to relevant networks)	3 (2–3)	24 (20.5)	75 (64.1)	18 (15.4)	*–*
Engage champions or opinion leaders (e.g. directors, managers) to assist with sharing of research findings	2 (2–3)	19 (16.2)	78 (66.7)	20 (17.1)	*–*
Engaging with social media (e.g. Facebook, Twitter)	2 (1–3)	35 (29.9)	61 (52.1)	20 (17.1)	*1 (0.9)*
Organising a media release/outreach campaign	2 (1–3)	48 (41.0)	57 (48.7)	8 (6.8)	*4 (3.4)*

^a^Rated as never (1) to always (5). For frequencies, “frequently” and “always”, and “rarely” and “occasionally” were pooled together

Comparative analysis showed significant differences in dissemination activities by experience, work setting, and KT training, but not research type. More experienced researchers reported using detailed reports (3.46 ± 0.99 vs. 3.02 ± 1.27, *t*(109) = − 2.01, *p* = 0.047) and developing new education materials (3.20 ± 0.86 vs. 2.76 ± 1.07, *t*(110) = − 2.38, *p* = 0.019), to a greater extent than less experienced researchers. A significant difference was found in publishing in peer-reviewed journals by work setting, *F*(2,71.55) = 27.13, *p* < 0.001, with researchers working in an academic setting (4.78 ± 0.42) reporting using this more frequently than those working in a joint work setting (4.27 ± 0.76) or an applied work setting (3.54 ± 1.13), all *p*’s < 0.05. Further, a significant difference was found in academic conference presentations by work setting, *F*(2,70.28) = 7.80, *p* < 0.001, with researchers working in applied work settings (3.57 ± 1.01) reporting using this method of dissemination less frequently than those working in an academic setting (4.26 ± 0.59, *p* < 0.001) or a joint work setting (4.09 ± 0.77, *p* = 0.020). However, no significant difference in dissemination through presentation at academic conferences was observed between researchers working in academic and joint settings (*p* = 0.606).

Several significant differences were observed in the use of dissemination activities between those who received KT training and those who did not. In particular, researchers reporting KT training more frequently developed new educational materials/sessions (3.31 ± 0.79 vs. 2.76 ± 1.05, *t*(114) = 2.78, *p* = 0.006), prepared a policy or evidence brief (3.03 ± 0.73 vs. 2.62 ± 0.96, *t*(91.35) = 2.52, *p* = 0.013), organised an interactive small group meeting/workshop (3.14 ± 0.76 vs. 2.76 ± 1.02, *t*(88.83) = 2.20, *p* = 0.030), organised a media campaign (2.36 ± 0.87 vs. 1.70 ± 0.92, *t*(111) = 3.62, *p* < 0.001), networked with end-users (2.86 ± 0.92 vs. 2.38 ± 1.06, *t*(115) = 2.42, *p* = 0.017), and engaged champions to share research findings (2.97 ± 0.87 vs. 2.31 ± 0.99, *t*(115) = 3.49, *p* < 0.001).

### Level of end-user engagement

Table 4 shows the self-reported level of end-user engagement. Most participants had engaged end-users in their research (87%). Participants reported that their engagement with end-users was mainly centred around informing them about findings through presentations, meetings, plain language summaries, or research papers, although 72% reported engaging end-users in their research beyond these activities. Almost half of the participants had consulted end-users about a research component or involved them directly throughout the research process. A quarter of participants reported having partnered with end-users in each aspect of the research. A small proportion of participants reported conducting end-user-initiated research.

**Table 4:** Level of end-user engagement (*n* = 117)

Level of engagement	*n* (%)
Letting them know about your research findings	70 (62.5)
Sent them my research papers	34 (30.4)
Sent them evidence briefs or plain language summaries	38 (33.9)
Presented my research to them	49 (43.8)
Held meetings, roundtables, or forums to discuss my research	41 (36.6)
Obtaining their feedback or input in any component of research	53 (47.3)
Working directly with end-users throughout the research process to ensure that concerns and aspirations are consistently understood and considered to the maximum extent possible	50 (44.6)
Partnering with end-users (i.e. shared decision-making) in each aspect of the research process	31 (27.7)
End-user-initiated research	19 (17.0)
I have not engaged end-users in my research	15 (13.4)

Significant differences were found in end-user engagement by career stage, work setting, and KT training. In particular, a greater proportion of established career researchers engaged end-users in their research beyond dissemination compared to early/mid-career researchers (82% vs. 65%, *χ*^2^(1) = 4.01, *p* = 0.045), with significant differences also found between researchers in a joint work setting (88%) compared to an applied work setting (71%) or an academic work setting (52%, *χ*^2^(2) = 12.33, *p* = 0.002), and between researchers with KT training (86%) compared to those without (65%, *χ*^2^(1) = 5.77, *p* = 0.016). Further, a greater proportion of researchers working in a joint work setting (55%) reported partnering with end-users compared to an academic work setting (30%) or an applied work setting (8.9%, *χ*^2^(2) = 22.22, *p* < 0.001). In addition, a significant difference in partnering with end users was also found in researchers with KT training (41%) compared to those without (21%, *χ*^2^(1) = 4.74, *p* = 0.030). Finally, a greater proportion of researchers in an academic work setting (33%) reported not engaging end-users in their research compared to researchers in applied work settings (11%) or joint work settings (3.0%, *χ*^2^(2) = 12.33, *p* = 0.002). No significant differences in end-user engagement were found by research type.

### Specific end-user groups and research stages

Follow-up questions were asked of those who reported engaging end-users in their research (*n* = 84) to determine which end-user groups they engaged and at what stage in the research process (see Table 5). The most common groups involved in research were frontline staff (80%) and senior management/policy-makers (79%), followed by blood donors/recipients (58%) and the general public (23%). Participants reported having experience engaging end-users throughout all of the research phases, with the most frequently reported phase being data collection (68%), followed by input into the study design and determining future research priorities stemming from results (both 56%). The least reported phases were data analysis (27%) and evaluation of research processes (23%).

**Table 5:** Engagement phase (*n* = 84)^a^

Research phase	*n* (%)
**End-user groups**	
Blood donors/recipients	49 (58.3)
Front-line staff	67 (79.8)
Senior management/policymakers	66 (78.6)
General public	19 (22.6)
Other	9 (10.7)
**Research phase**	
Research priority-setting	40 (47.6)
Grant proposal/protocol writing	42 (50.0)
Input into methodology/study design	47 (56.0)
Development of research questions	45 (53.6)
Data collection	57 (67.9)
Data analysis	23 (27.4)
Interpretation of results	37 (44.0)
Input into the selection of research translation products	27 (32.1)
Evaluation of research processes	19 (22.6)
Determining future research priorities stemming from results	47 (56.0)

^a^Asked only to those who indicated engaging end-users in their research. Multiple choice

## Discussion

Translating research is seen as important by transfusion medicine researchers, with most considering it their responsibility to ensure that their research is translated. However, many researchers feel they do not have skills or knowledge of strategies to translate the knowledge gained from their research. Researchers typically focus on sharing their knowledge through traditional diffusion strategies, with more tailored dissemination approaches used to a lesser extent. Further, whilst most participants had informed end-users of their research findings, only half of the sample also had experience with consulting end-users about a research component or involving them throughout the research process. Only slightly more than 1 in 4 researchers in this study reported an experience working in genuine partnership with end-users and only 1 in 6 had conducted end-user-initiated research. These findings are aligned with other studies [12–16] conducted in other health-related areas where traditional diffusion strategies were more frequently used than tailored approaches.

However, our study did find differences in the use of dissemination strategies. Training in KT was found to be associated with greater perceived KT skills and knowledge of KT strategies. It was also associated with greater use of tailored dissemination strategies, such as developing new educational materials/sessions and small group meetings or workshops, and end-user engagement activities, such as partnering with end-users, compared to those without. The benefits of KT training were also documented in a recent study where trainees had greater knowledge of KT, perceived skills to practice KT, and greater perceived ability to engage with end-users after receiving KT training [29]. This suggests that providing KT training to transfusion medicine researchers may be an effective strategy to increase KT in this area. Whilst this difference may be attributed to researchers with an interest in KT undergoing training, many of our surveyed sample identified that they would like to have access to KT education and training [25]. Further research is needed to develop and evaluate a KT training programme for transfusion medicine researchers as a way to increase their knowledge, confidence, and use of KT activities.

Our research also identified a difference in KT views and activities by career stage. Established researchers reported greater knowledge of KT strategies, skills to facilitate KT, and available funding for KT than less experienced researchers. This discrepancy in abilities and resources may have affected KT practices, with established researchers having written detailed reports, developed new education materials, and engaged end-users in their research to a greater extent than early/mid-career researchers. A potential explanation for this finding may be that researchers working in transfusion medicine for a longer period of time have had the opportunity to conduct more research and therefore have had a greater need for knowledge to be translated compared to researchers relatively new to the area. Further, they may have had more time to form connections with end-users and gain experiential knowledge on effective KT strategies as no significant differences were found between the two groups in self-reported KT training. It is important that this knowledge is shared with early and mid-career researchers to support their KT efforts through for example mentoring or collaboration through facilitated networks [30–32]. It is recommended that these knowledge sharing strategies are further investigated.

Another factor that appears to affect KT practice is the setting in which the researcher is located. We found that researchers working solely in an academic setting reported more traditional diffusion strategies and less end-user engagement activities than researchers working (to some extent) in an applied setting. There are several possible explanations for this finding. Some academic institutions may place a greater emphasis on traditional diffusion methods, such as peer-reviewed publications, as performance indicators and considerations for promotion. In contrast, health services may place a greater value on research that leads to improved outcomes for their patients, blood donors, staff, or the health service itself [33]. Further, funding may affect the type and topic of the research conducted; researchers working in applied settings are often funded directly by the blood collection agency or health service who desire practical solutions to their issues [22]. In contrast to health services, external funding bodies may place more emphasis on traditional diffusion strategies [34, 35]. Finally, researchers working in an applied or joint position within a blood collection agency or other health service may have had more opportunities to create end-user networks and find it easier to engage with these networks throughout the research process. As a result, the knowledge generated through the research may be more directly relevant to issues faced by the blood collection agency or health service and more easily translatable to policy and/or practice [5, 10, 11, 36].

Of equal interest is the limited differences observed between basic and applied science researchers. In our study, with the exception of basic science researchers reporting greater KT skills, no differences were found between basic science researchers and applied science researchers in their views of KT, how they share their knowledge, and the extent they engage end-users in their research. This is somewhat surprising as the literature has suggested that the purpose of KT differs between the two groups. Basic science researchers are assumed to focus on translation to clinical science and knowledge, with outcomes such as clinical use or commercialisation of new treatments. On the other hand, applied sciences are assumed to focus on translation to healthcare and services, with outcomes such as treatments are being used appropriately [19, 20]. However, our findings align with the experiences of stroke rehabilitation researchers, in which pre-clinical and clinical researchers reported similar research translation views and practices [28]. This suggests that, whilst their KT purpose may differ, basic and applied science researchers apply similar KT dissemination and end-user engagement activities.

### Limitations

There were several limitations to the study. First, researchers with no interest in KT may have opted out of participating affecting the generalisability of the results. Second, the sample size was relatively small in comparison with the number of survey invitations sent directly to corresponding authors and grant recipients as well as likely views of the social media posts. However, our sample was diverse in the type of research, work setting, location, career stage, and self-reported KT training suggesting our insights reflect the broader transfusion medicine research community. Third, our sample may include research trainees as we did not screen for this in our survey. Whilst this may have influenced some of our findings regarding less experienced researchers, our recommendation for the need to better support less experienced researchers through sharing knowledge of established researchers remains. Fourth, the study materials, including the questionnaire, were only presented in English, which may have limited our sample to researchers fluent in English. Nevertheless, our sample does include participants from a wide range of countries. Fifth, we only focused on two aspects of KT, and further research is needed to examine researchers’ practices relating to the synthesis and application of knowledge. Finally, KT activities were self-reported and assessed over their career in general as a transfusion medicine researcher, which may have led to some recall bias. In addition, it may have also led to social desirability bias causing overreporting of KT activities. Future research could look to measuring KT activities more objectively.

## Conclusions

This study showed that transfusion medicine researchers consider KT as being important and feel it is part of their responsibility. However, there appear to be gaps in their knowledge and limited support to conduct KT. Our work highlights that KT knowledge needs to be shared across all health-related areas, including transfusion medicine, to ensure knowledge producers, such as researchers, can benefit from advancements made in the field of KT and implementation science.

### Supplementary Information

**Additional file 1.** STROBE Statement—checklist of items that should be included in reports of observational studies.

**Additional file 2.** Questionnaire Knowledge Translation in Transfusion Medicine.

## Data Availability

The datasets generated and/or analysed during the current study are not publicly available due to privacy restrictions but may be available from the corresponding author upon reasonable request, subject to ethics and institutional approval.

## Abbreviation

KT: Knowledge translation
