# Supplementary material for: Researchers’ views on and practices of knowledge translation: an international survey of transfusion medicine researchers
Source: Implement Sci Commun. 2024 Jan 12;5:9. doi: 10.1186/s43058-024-00546-3 (PMC10787432; doi:10.1186/s43058-024-00546-3)
Supplement: Supplementary file 2 — Additional file 2. Questionnaire Knowledge Translation in Transfusion Medicine. [file 43058_2024_546_MOESM2_ESM.docx]

**Questionnaire Knowledge Translation in Transfusion Medicine**

**Demographic and professional characteristics**

These first set of questions will ask about your demographic and professional background.

In which country are you based? _______________________

How do you describe your gender?

- Man/Male
- Woman/Female
- Non-binary
- I use a different term
- Prefer not to say

Which best describes the setting in which you work?

- University
- Research institute (not within a university)
- Government department or agency
- Blood collection agency
- Hospital setting
- Healthcare service (not a blood collection agency or hospital)
- Other

Please specify other work setting: ______________________

Do you also currently work in any of the other settings?

- Yes, namely [*insert dropdown with previous question options*]
- No

What type of research are you currently using? Please select all that apply:

- Animal studies
- Biospecimen analysis research
- Data linkage research
- Epidemiological research
- Interventional/Clinical trials research
- Qualitative research
- Quantitative research
- I do not conduct research
- Other

How many years have you worked within the area of transfusion medicine? ____________________

Have you ever received training on translating research into policy and/or practice (i.e. knowledge translation)?

- Yes
- No
- Unsure/Don't know

**Knowledge translation activities**

These next set of questions will ask you about any activities you may undertake to share your research findings, and the possible response categories range from *never* to *always*. When answering these questions, please keep in mind that how often you undertook each activity may depend on how often it was feasible for you to do so, given the nature of the activity and the context in which you work.

- If you undertook a particular activity whenever it was feasible to do so, please indicate:
  - *always* if you undertook the activity every single time it was feasible or
  - *frequently* if you did so almost every single time it was feasible.
- If you undertook a particular activity at least once but much less often than it was feasible to do so, please indicate:
  - *occasionally* if you undertook the activity more often than not or
  - *rarely* if you hardly ever did so.
- If you never undertook a particular activity whether it was feasible to do so or not, please indicate *never*.

**Diffusion activities**

To what extent do you do the following activities to disseminate your research findings? (1=Never, 2=Rarely, 3=Occasionally, 4=Frequently, 5=Always)

- Publishing in peer reviewed journals
- Presenting at an academic conference
- Detailed research reports

**Dissemination activities**

To what extent do you do the following activities to disseminate your research findings? (1=Never, 2=Rarely, 3=Occasionally, 4=Frequently, 5=Always)

- Developing new educational materials/sessions
- Preparing policy or evidence brief and disseminating them to relevant audiences (e.g., policy-makers, health service providers or administrators)
- Organising interactive small group meeting/workshop
- Writing plain language summaries
- Engaging with social media (e.g. Facebook, Twitter)
- Organising a media release/outreach campaign
- Creating networks or networking with end-users such as policy-makers and practitioners (e.g., give presentations to relevant networks)
- Engage champions or opinion leaders (e.g., directors, managers) to assist with sharing of research findings

**End-user engagement activities**

This section asks about your knowledge and experience with engaging potential end-users (e.g., policy makers, blood processing staff, hospitals, donors, recipients) in research.

At what level have you engaged end-users (e.g., policy makers, blood processing staff, hospitals, donors, recipients) in your research? *[multiple choice]*

- Letting them know about your research findings.
- Obtaining their feedback or input in any component of research.
- Working directly with end-users throughout the research process to ensure that concerns and aspirations are consistently understood and considered to the maximum extent possible.
- Partnering with end-users (i.e. shared decision-making) in each aspect of the research process.
- End-user initiated research.
- I have not engaged end-users in my research. *[exclusive option > skip to next block]*

[*If selected “Letting them know about your research findings”*] How did you let them know about your research findings? [*multiple choice*]

- Sent them my research papers
- Sent them evidence briefs or plain language summaries
- Presented my research to them
- Held meetings, roundtables or forums to discuss my research

Who have you engaged in the research process? Please select all that apply:

- Blood donors
- Blood recipients
- Blood collection staff
- Blood processing staff
- Senior management
- Policy makers
- Hospital staff
- General public
- Other, namely: _____

There are different points in the research process where end-users (e.g., policy makers, blood processing staff, hospitals, donors, recipients) could potentially be engaged. Please indicate those research phases where you have experience engaging with end-users. Please select all that apply:

- Research priority-setting
- Grant proposal/protocol writing
- Input into methodology/study design
- Development of research questions
- Data collection
- Data analysis
- Interpretation of results
- Input into the selection of research translation products
- Evaluation of research processes
- Determining future research priorities stemming from the results
- Other, please specify….

**Importance and responsibility for knowledge translation**

We would like to know more about your views on who should be responsible for and the importance of translating research into policy and/or practice.

Please rate your agreement with the following statements: (1=strongly disagree, 2=disagree, 3=neither agree not disagree, 4=agree, 5=strongly agree)

1. It is important to me that my research is translated.
2. My research is not the sort of research that can be translated.
3. It is my responsibility to ensure that my research is translated.
4. Research translation is the responsibility of someone else in my team
5. Researchers should be responsible for translating findings into practice.
6. Stakeholders should be responsible for translating research findings into practice.
7. I know which strategies should be used (by myself/others) to translate research.
8. I have the skills to ensure research is translated.
9. There is adequate funding available to support translation of research.
10. Spending time on translating my research would take me away from research or other work-related activities I enjoy.
11. Researchers with experience/interest in implementation should translate research.
12. Every research team should include a researcher with expertise in implementation.
